# Smoking, physical activity and healthy aging in India

**DOI:** 10.1186/1471-2458-14-526

**Published:** 2014-05-29

**Authors:** Jane Murray Cramm, Jinkook Lee

**Affiliations:** 1 Institute of Health Policy and Management, Erasmus University, Rotterdam, The Netherlands; 2 Professor, Davis School of Gerontology, University of Southern California, Los Angeles & Senior Economist, RAND Corporation, Santa Monica, California, United States of America

**Keywords:** Community-dwelling older people, Healthy aging, India, Physical activity, Smoking

## Abstract

**Background:**

To identify levels of physical inactivity and smoking and examine their relationships to health among older people in India.

**Methods:**

In 2010, Longitudinal Aging Study in India researchers interviewed 1,683 older adults in randomly sampled households with members aged ≥ 45 years in eight stratified districts in four states (90.9% response rate). We first used descriptive analyses to characterize older people in poor and good health. Differences between groups were established using chi-squared and *t*-tests. Multivariate logistic regression analyses were then performed to determine whether physical inactivity and smoking led to poor health while controlling for district of residence, caste, age, gender, marital status, and educational level. Regression analyses were also used to identify significant relationships between socio-demographic characteristics and health behaviors.

**Results:**

Larger proportions of older people in poor health were smokers (26.1% *vs.* 16.9%; *p* ≤ 0.001) and physically inactive (vigorous activities: 88.7% *vs.* 70.7%, *p* ≤ 0.001; moderate activities: 67.1% *vs.* 57.1%, *p* ≤ 0.01). Smoking (*p* ≤ 0.05) and lack of vigorous physical activity (*p* ≤ 0.001) increased the likelihood of poor health. Low educational level was significantly related to smoking and the lack of moderate physical activity (both *p* ≤ 0.001). Female gender decreased the likelihood of smoking. Male gender increased the likelihood of vigorous physical activity but decreased the likelihood of moderate physical activity.

**Conclusions:**

Smoking and physical inactivity have important impacts on the health of older people in India. Policy attention is needed to improve these modifiable health behaviors.

## Background

India’s population is aging rapidly; increased life expectancy and a decline in fertility have dramatically changed its age composition. The proportion of individuals aged ≥ 60 years will grow almost threefold (from 8.4% to 22.6%), and that of individuals aged ≥ 80 years will increase nearly fourfold (from 0.8% to 3%) [1]. With population aging and economic development, the disease profile of the country has shifted from predominantly infectious to chronic diseases. The country now faces an enormous burden of chronic conditions, and the situation is expected to worsen in the future [2]. Chronic diseases are a major cause of morbidity among India’s older inhabitants; they were responsible for 53% of deaths and 44% of disability-adjusted life years lost [3]. India ranks second among the top 10 countries in the prevalence of people with diabetes [4], and this prevalence is expected to increase 150.5%, from 31.7 million to 79.4 million, between 2000 and 2030 [5].

The continued increase in life expectancy is a major achievement, and keeping older people active and healthy, rather than the aging of the population *per se*, constitutes a challenge. The rapid increase in the prevalence of chronic illness resulting from unhealthy lifestyles has increased the demand for health care services and constrained the organization and delivery of care [6-8]. Health behaviors such as smoking and physical inactivity are risk factors for many chronic diseases and leading causes of death and disability [9]. Thus, improving health behaviors to prevent the onset of chronic diseases is becoming a critical issue [10,11].

Given the growing prevalence of chronic diseases in older populations, constraints on health care expenditure and the prevention of chronic illnesses and disabilities have been identified as serious public health problems [12]. Prevention of these conditions and improving health behaviors are thus considered priorities for geriatric research and clinical practice [13].

In India, smoking is more prevalent in men than in women and among older people [14]. Whereas men smoke throughout their lives, women tend to become smokers at an older age [15]. Furthermore, Bhan and colleagues [16] found that smoking was more prevalent among less-educated, poorer, rural, and lower-caste men, and among women in urban areas. They also documented a greater decline in smoking over time among more-educated women [16]. High levels of physical inactivity have also been reported for the Indian population [17], and inactivity has been found in greater proportions of urban than rural populations [18].

Although some studies have investigated smoking or physical activity, little research has examined both health behaviors and their relationships to health in older people. Given the need for healthy aging in India’s older population, this study thus aimed to (i) identify levels of physical inactivity and smoking among older people in India, (ii) examine the relationships between these behaviors and the likelihood of reporting poor self-stated health in this population, and (iii) investigate relationships between socio-demographic characteristics and health behaviors.

## Methods

The Longitudinal Aging Study in India (LASI) was designed to examine a nationally representative sample of India’s population aged ≥ 45 years. LASI is a partnership among the Harvard School of Public Health (HSPH), the International Institute for Population Sciences (IIPS), and the RAND Corporation. The study is gathering data on the social, economic, and health situations of older people throughout India with the goal of providing policymakers with information needed to improve health and health behaviors in this segment of the population. For the pilot study, a representative sample of 1,683 individuals was interviewed in four states in October 2010. Detailed descriptions of the study design and methodology can be found in Arokiasamy *et al.*[19] and Chien *et al.*[20].

### Sample

The pilot study was conducted in four Indian states—Rajasthan and Punjab in the north and Kerala and Karnataka in the south—and fielded in the dominant language of each state. These states were chosen to capture the demographic, economic, health, and cultural diversity of India. The sampling plan was based on the 2001 Indian Census. Eight primary sampling units (districts) were chosen to be surveyed. These districts were stratified across urban and rural areas within each state to represent a variety of socioeconomic conditions. Eligible households were defined as those with at least one member aged ≥ 45 years. Eligible individuals in these households were aged ≥ 45 years or married to an individual of that age. LASI researchers randomly sampled 1,546 households in these stratified districts, and conducted interviews with 1,683 members of 950 households in October to December 2010.^19^ The LASI achieved a household response rate of 88.5% and an individual response rate of 90.9%.

### Ethical approval and informed consent

Institutional review boards at Harvard University and the IIPS approved the study protocol, and informed consent was obtained from all study participants.

### Outcome measure

This study measured self-rated health, which is considered to be a valid and robust measure of general health status. A large body of evidence has demonstrated that self-reported health assessment has high predictive validity for mortality, physical disability, and chronic disease status. Self-assessed health has also been shown to be a stronger predictor of mortality than physician-assessed health [21-23]. In this study, respondents were asked to rate their perceived health on a five-point ordinal scale with the question: ''How would you describe your overall state of health these days? Would you say it is (5) excellent, (4) very good, (3) good, (2) fair, or (1) poor?'' This outcome variable was dichotomized into poor (1, 2) and good (3–5) responses.

### Socio-demographic measures

We included the following socio-demographic variables in analyses: geographic area (state, urban or rural), caste, age, gender, marital status, and education. Older people’s educational levels were assessed using six categories ranging from 0 (no schooling) to 5 (more than high school). We dichotomized this item into low (primary school or less; 1) and high (completed middle school or more; 0) categories of education. Castes were classified as scheduled castes, scheduled tribes, and non-scheduled castes or tribes. Scheduled castes and tribes are historically disadvantaged social classes in India.

### Health behavior measures

Self-reported current smoking was assessed with a yes/no question.

Vigorous and moderate physical activity was assessed by asking respondents how often they were physically active; responses were structured as 1 (“every day”), 2 (“more than once a week”), 3 (“once a week”), 4 (“one to three times per month”), and 5 (“hardly ever or never”). To assess vigorous activity, respondents were asked how often they took part in sports or activities such as running or jogging, swimming, going to a health center or gym, cycling, digging with a spade or shovel, heavy lifting, chopping, farm work, fast bicycling, or cycling with loads. To assess moderate physical activity, respondents were asked how often they took part in activities such as cleaning house, washing clothes by hand, gardening, bicycling at a regular pace, walking at a moderate pace, dancing, or floor or stretching exercises. Asking about people’s physical activities is considered to be sufficiently reliable and valid to measure the level of physical activity in a healthy adult population [24] and to assess physical activity in patients after total hip arthroplast [25]. Based on responses to items about vigorous and moderate physical activity, we classified respondents as physically inactive (engaging in physical activity once a week or less often) and physically active (engaging in physical activity more than once a week). The LASI questionnaire was designed with the harmonization with other aging surveys, such as the Health and Retirement Study in the United States and the China Health, Aging, and Retirement Longitudinal Study, as a goal to enable cross-country studies, and the questions on smoking and physical activities were adopted from these sister studies and modified to the Indian context (for example, listing various types of vigorous physical activities common in India).

### Statistical analyses

We first used descriptive analyses to characterize older people in poor and good health. Differences between groups were established using chi-squared and *t*-tests. Multivariate logistic regression analyses were then performed to determine whether physical inactivity and smoking led to poor health while controlling for district of residence, caste, age, gender, marital status, and educational level. Regression analyses were also used to identify significant relationships between socio-demographic characteristics and health behaviors. SPSS software (version 20; IBM) was used for statistical analyses.

## Results

Table 1 summarizes the demographic characteristics of this sample of older people in India. Mean age of respondents is 55.8 years (standard deviation of 11.9), of which 30% are 60 years of age or older. More older adults in urban and rural areas of Kerala were in poor health, whereas larger proportions of older adults in good health resided in Rajasthan (rural) and Karnataka (urban and rural). Age and marital status differed between older people in poor and good health. A significantly larger proportion of older people in poor health was single (40.5% *vs.* 16.3%; *p* ≤ 0.001), and those in poor health were, on average, significantly older than older people in good health (65.7 *vs.* 54.2 years; *p* ≤ 0.001).

**Table 1:** Demographic characteristics of older people in poor and good health in India

**Characteristic**	**Total**	**Poor health**	**Good health**	***χ***^**2**^	***t***	***p***^**†**^
	***n*** **= 1679**	***n*** **= 222 (13.2%)**	***n*** **= 1457 (86.8%)**			
Punjab (urban)	6.9%	5.4%	7.1%	0.90		0.40
Punjab (rural)	17.0%	14.4%	17.4%	1.24		0.29
Rajasthan (urban)	5.2%	4.5%	5.4%	0.28		0.75
Rajasthan (rural)	19.5%	18.5%	19.8%	0.21		0.72
Kerala (urban)	8.0%	13.1%	7.1%	9.27		≤0.01
Kerala (rural)	19.5%	33.8%	17.2%	33.75		≤0.001
Karnataka (urban)	8.0%	1.8%	8.9%	13.30		≤0.001
Karnataka (rural)	15.9%	8.6%	17.0%	10.32		≤0.001
Scheduled castes	91.8%	95.5%	91.2%	4.70		≤0.05
Scheduled tribes	5.4%	2.7%	5.8%	3.70		0.06
Non-scheduled castes/tribes	2.8%	1.8%	3.0%	0.94		0.51
Gender (female)	56.4%	59.9%	55.7%	1.38		0.25
Age [years; mean (SD)]	55.8 (11.9)	65.7 (12.8)	54.2 (11.0)		12.62	≤0.001
Marital status (single)	19.4%	40.5%	16.3%	72.38		≤0.001
Educational level (low)	69.8%	74.2%	69.2%	2.30		0.14
Smoking (yes)	18.1%	26.1%	16.9%	11.0		≤0.001
Physical inactivity^‡^ (vigorous)	73.0%	88.7%	70.7%	31.86		≤0.001
Physical inactivity (moderate)	58.4%	67.1%	57.1%	8.03		≤0.01

^†^*p* indicates differences between groups; ^‡^physical inactivity was defined as engagement in activities once a week or less often. SD, standard deviation.

Survey responses indicated that 18.1% of study participants were current smokers, and 73.0% and 58.4% engaged in vigorous and moderate physical activities, respectively, once a week or less often. Compared with those in good health, significantly larger proportions of older people in poor health were smokers (26.1% *vs.* 16.9%; *p* ≤ 0.001) and physically inactive (vigorous activities: 88.7% *vs.* 70.7%; *p* ≤ 0.001; moderate activities: 67.1% *vs.* 57.1%; *p* ≤ 0.01).

Multivariate logistic regression analyses indicated that older age (*p* ≤ 0.01) and being single (*p* ≤ 0.001) were related to a higher likelihood of poor health (Table 2). Compared with the reference area (rural Kerala), living in Punjab (urban and rural areas) or Rajasthan (urban and rural) decreased the likelihood of poor health and living in Karnataka (urban and rural) increased this likelihood. Smoking (*p* ≤ 0.05) and physical inactivity in the vigorous category (*p* ≤ 0.001) were found to increase the likelihood of poor health among older people in India in analyses that controlled for important background characteristics.

**Table 2:** Relationships between socio-demographic characteristics and health behaviors and health outcomes among older people in India

	**Poor health *n*** **= 1672**
	**Adjusted odds ratio**
Punjab (urban)	0.417 (0.200–0.868)^*^
Punjab (rural)	0.414 (0.239–0.719)^**^
Rajasthan (urban)	0.433 (0.197–0.949)^*^
Rajasthan (rural)	0.498 (0.290–0.855)^**^
Kerala (urban)	0.938 (0.543–1.622)
Karnataka (urban)	0.107 (0.037–0.310)^***^
Karnataka (rural)	0.279 (0.151–0.514)^***^
Tribe	0.505 (0.199–1.279)
No caste or tribe	0.393 (0.129–1.197)
Gender (female)	1.257 (0.855–1.848)
Age	1.059 (1.043–1.074)^**^
Marital status (single)	1.727 (1.181–2.527)^***^
Educational level (low)	1.307 (0.861–1.984)
Smoking (yes)	1.610 (1.048–2.474)^*^
Physical inactivity^†^ (vigorous)	2.437 (1.508–3.937)^***^
Physical inactivity (moderate)	1.219 (0.820–1.811)
Model *χ*^2^	224.006^***^
−2 log likelihood	1081.840
Nagelkerke *R*^2^	0.231

^†^Physical inactivity was defined as engagement in activities once a week or less often. ^*^p ≤ 0.05, ^**^p ≤ 0.01, ^***^p ≤ 0.001. Reference groups for region and caste/tribe affiliation were Kerala (rural) and caste, respectively (largest groups).

In analyses examining the relationships between background characteristics and health behaviors, we found that educational level was significantly related to smoking and moderate physical activity (both *p* ≤ 0.001; Table 3). Larger proportions of less-educated people smoked and were physically inactive. We also found significant relationships between gender and health behaviors. Female gender decreased the likelihood of smoking. Relationships between gender and physical inactivity were mixed; female gender increased the likelihood of physical inactivity in the vigorous category but decreased this likelihood in the moderate category. Furthermore, tribe members smoked less but engaged less in moderate and vigorous physical activities compared with people in higher castes.

**Table 3:** Relationships between socio-background characteristics and health behaviors among older people in India

	**Smoking *n*** **= 1675**	**Physical inactivity (vigorous) *n*** **= 1675**	**Physical inactivity (moderate) *n*** **= 1683**
	**Adjusted OR**	**Adjusted OR**	**Adjusted OR**
Punjab (urban)	0.060 (0.022–0.159)^***^	2.220 (1.303–3.781)^**^	7.738 (4.600–13.017)^***^
Punjab (rural)	0.042 (0.021–0.085)^***^	2.081 (1.397–3.100)^***^	4.533 (3.113–6.602)^***^
Rajasthan (urban)	0.329 (0.160–0.677)^**^	2.758 (1.472–5.167)^**^	11.167 (6.075–20.527)^***^
Rajasthan (rural)	0.298 (0.182–0.486)^***^	1.260 (0.837–1.896)	4.479 (2.991–6.709)^***^
Kerala (urban)	0.972 (0.568–1.662)	1.175 (0.733–1.884)	0.647 (0.398–1.053)
Karnataka (urban)	0.370 (0.204–0.672)^***^	1.966 (1.203–3.212)^**^	4.422 (2.825–6.921)^***^
Karnataka (rural)	0.564 (0.354–0.899)^*^	1.623 (1.086–2.427)^*^	3.295 (2.242–4.844)^***^
Tribe	0.476 (0.227–0.995)^*^	1.824 (1.031–3.227)^*^	2.220 (1.213–4.062)^**^
No caste or tribe	1.221 (0.541–2.757)	0.655 (0.342–1.256)	0.281 (0.140–0.566)^***^
Gender (female)	0.075 (0.052–0.110)^***^	3.317 (2.574–4.273)^***^	0.601 (0.473–0.765)^***^
Age	1.009 (0.996–1.024)	0.979 (0.691–1.388)^***^	1.062 (0.776–1.454)^***^
Marital status (single)	1.351 (0.873–2.088)	1.050 (1.037–1.062)	1.043 (1.032–1.055)
Educational level (low)	2.609 (1.784–3.814)^***^	0.777 (0.583–1.037)	1.594 (1.212–2.095)^***^
Model *χ*^2^	420.380^***^	160.087^***^	374.369^***^
−2 log likelihood	1163.329	1793.333	1899.906
Nagelkerke *R*^2^	0.363	0.132	0.270

^*^p ≤ 0.05, ^**^p ≤ 0.01, ^***^p ≤ 0.001. Reference groups for region and caste/tribe affiliation were Kerala (rural) and caste, respectively (largest groups). OR, odds ratio.

## Discussion

The results of this study revealed pronounced physical inactivity among older people in India, and demonstrated that smoking and physical activity were significantly related to health in this population. These results support prior evidence that physically active older men and women have lower rates of all-cause mortality, coronary heart disease, high blood pressure, stroke, type 2 diabetes, and cancer, and higher levels of cardiorespiratory and muscular fitness, and healthier body mass and composition, compared with less active older people [26]. Furthermore, the biomarker profile of physically active older people is more favorable for the prevention of cardiovascular disease and type 2 diabetes and the enhancement of bone health, and they exhibit higher levels of functional health, a lower risk of falling, better cognitive function, and reduced risks of moderate and severe functional and role limitations [26,27].

The World Health Organization (WHO) has recommended that older people engage in at least 150 minutes of physical activity per week, e.g., 30 minutes of activity 5 times per week. This recommendation applies to all individuals aged ≥ 65 years, irrespective of gender, race, ethnicity, or income level. It also applies to older people with chronic diseases. In contrast, our findings suggest that a majority of older Indians are physically inactive – only 37% of men and 19% of women engaged in vigorous activities more than once a week, and 48% of men and 34% of women engaged in moderate activities more than once a week. The WHO stated that inactive older people will gain additional health benefits by shifting from complete inactivity to some level of activity [28]. Given the growing prevalence of chronic diseases in the aging population of India, efforts to prevent chronic diseases and improve health behaviors, especially physical activity, are needed.

We found that older people in poor health reported more physically inactivity (moderate and vigorous) and that more were smokers than those in good health. Although no direct relationship was found between educational level and health outcomes, our research identified significant relationships between educational level and smoking and engagement in moderate physical activity. Similar to the findings of Bhan and colleagues [16], this study showed that larger proportions of less-educated people smoked compared with more-educated people. In addition, this study revealed more physical inactivity among less-educated people. These relationships between education and health behaviors contribute to education-related gradients in health in India.

We also found significant relationships between gender and health behaviors. Like previous studies [14,16], we found that women were less likely than men to smoke. The relationships between gender and physical inactivity were mixed. Men reported engaging more often in vigorous activities such as sports, whereas women reported more engagement in moderate physical activities, such as cleaning house, washing clothes by hand, and gardening. This finding is not unexpected, given that the traditional role of women in India is confined to the household.

This study has several limitations. The cross-sectional design hampered our abilities to draw causal conclusions and investigate longitudinal relationships over time. When longitudinal data become available, we will investigate the longitudinal effects of (changes in) health behavior in India’s older population. For example, the relationship between physical activity and health is expected to be dynamic: physical inactivity will lead to poor health outcomes, but poor health will also lead to physical inactivity. Longitudinal data would have enabled us to disentangle the dynamic relationship between physical activity and health. Furthermore, this study did not include the effects of health behaviors on clinical (objective) outcomes. Other research did, however, show that smoking was related to a significantly higher probability of elevated blood pressure, fasting plasma glucose, and metabolic syndrome in women [29]. In addition, Kwaśniewska and colleagues [29] found that physical activity is expected to reduce the prevalence of metabolic syndrome among never and past smokers. Future research is necessary investigating both subjective and objective measures and identify differences in their relationship with health behaviors. Goverover and colleagues [30], for example found that objective measures and subjective self-report measures of functional activities are not related to each other suggesting that they both assess different dimensions of health each with their own strengths and weaknesses. Recently, Cole [31] found that subjective measures of health are better predictors of disease and mortality since perceptions and social factors affect gene expression. Social stimuli and perceptions of social conditions seem to regulate the expression of neural genes such as the nerve growth factor gene [32] the glucocorticoid receptor gene [33] the key immune system genes [32] and leukocytes [33]. These findings show that individuals' perceptions of their health result in changes in their genes, where more positive subjective appraisals of health relate to more favorable clinical/objective health outcomes.

## Conclusions

We can conclude that health behaviors are related to health outcomes in India’s older population. Our findings reveal much room for improvement in older Indians’ health behaviors, especially regarding physical inactivity. Recognition of the relatively high levels of physical inactivity and smoking among people aged 45 years and older in India is an important reality check, and by linking physical activity and smoking behaviors to health, we call for putting physical activity and smoking cessation to high priority on public health agenda. As these health behaviors are modifiable, our findings have important implications for health policy in India. Efforts to reduce smoking among older men and to keep older people physically active are needed. Improved health behaviors are expected to improve health outcomes and prevent or delay the onset of disabilities and chronic diseases. Based on these findings, we recommend the development and implementation of educational and interventional programs to improve health behaviors. These programs may be customized for various groups (e.g., more- and less-educated people, men and women).

## Competing interests

The authors declare that they have no competing interests and confirm all patient/personal identifiers have been removed or disguised so the patient/person(s) described are not identifiable and cannot be identified through the details of the story.

## Authors’ contributions

JL drafted the design for data gathering and was involved in acquisition of subjects and data. JC and JL performed statistical analysis and interpretation of data. JC drafted the manuscript and JL helped drafting the manuscript and contributed to refinement. Both authors have read and approved its final version.

## Pre-publication history

The pre-publication history for this paper can be accessed here:

http://www.biomedcentral.com/1471-2458/14/526/prepub
